# Excessive active pharmaceutical ingredients in substandard and falsified drugs should also raise concerns in low-income countries

**DOI:** 10.7189/jogh.14.03029

**Published:** 2024-06-07

**Authors:** Elisa M Maffioli, Marie C Montás, Chimezie Anyakora

**Affiliations:** 1 University of Michigan, School of Public Health, Department of Health Management and Policy, Ann Arbor, Michigan, USA; 2 Harvard T. H. Chan School of Public Health, Department of Global Health and Population, Boston, Massachusetts, USA; 3 Bloom Public Health, Abuja, Nigeria; 4 School of Science and Technology, Pan Atlantic University, Lagos, Nigeria

Substandard and falsified (SF) medicines still do not receive enough attention in public health. Substandard medicines are authorised medical products that fail to meet their quality standards or specifications, or both, due to within-factory errors or degradation in supply chains. Falsified medicines, instead, are defined as deliberately and fraudulently misrepresenting medicines’ identity, composition, or source [1]. Due to the underground nature of the counterfeit drug business, SF medicines are hard to detect, and it is difficult to have representative figures on their prevalence [2]. SF medicines may be reported through recalls, warnings, alerts, and case reports of patients not responding to medication, the purpose of which is not to estimate the prevalence of the quality of individual drug samples. Most studies set up to this goal are cross-sectional and incorporate a range of sampling strategies to identify informal sellers of SF medicines in markets, including multistage, random, convenience or purposive sampling, and a mystery-shopper approach [3]. Despite limitations on these sample strategies, due to the high costs of larger random data collection, these methods remain the most used to identify SF medicines worldwide.

Despite the lack of accurate data, SF medicines are still a significant policy concern worldwide. In 2010, the United Nations (UN) Office on Drugs and Crime highlighted the trafficking of SF medical products as a substantial threat, alongside cocaine, maritime piracy, and human trafficking. The illegal trafficking of SF medicines remains the most profitable sector of the global trade in copied goods because it is low risk and has an innate capacity for demand. In low-income countries, SF medicines are even more of a concern, as outright corruption and weak institutions may limit the government’s capacity to address inefficiencies along the drug supply chain. Further, individuals are more financially constrained, limiting their choice of medicines when purchasing at a pharmacy.

The most comprehensive evidence on the prevalence of SF medicines in low-income countries is dated a few years back. In 2017, the World Health Organization (WHO) identified 100 published papers between 2007–13 reporting on the testing of 48 218 samples of medicines collected from 88 countries. It is estimated that in low- and middle-income countries (LMICs), 10.5% of medicines are low-quality [1]. A similar investigation of 265 studies comprising 400 647 drug samples and a meta-analysis of 96 studies comprising 67 839 drug samples estimated the prevalence of SF medicines in LMICs at 13.6% (19.1% for antimalarials and 12.4% for antibiotics) [4]. Several other studies analysed smaller samples from a smaller set of countries, adding evidence that the prevalence of SF medicines varies widely by country and type of drug. Sub-Saharan Africa is among the regions with the highest prevalence [5]. In general, antimalarials and antibiotics receive more attention [6,7].

Despite its nature, the counterfeit pharmaceutical trade is quite unequal. SF medicines account for up to 50% of sales in LMICs but only 1% in high-income countries. This phenomenon is happening despite the fact that the Middle East and African pharmaceutical markets represent 2.96% of the global market share, compared to 45.33% for North America [8]. In addition to methodological issues such as poor sampling techniques and analytical procedures in testing, several systemic factors contribute to this disparity between low and high-income countries [9]. Among others, poor manufacturing and inadequate quality-control processes in producing medicines may play a role. More broadly, compared to high-income countries, low-income countries have poorer regulatory frameworks and unmet market demands due to scarcity of specific drugs or high prices that open up a market for SF medicines.

Several initiatives and successes have addressed the problem of SF medicines [10]. First, to better understand the problem, there have been international and country-level initiatives for systematic collection and accurate, transparent documentation of information on drug manufacturers, including programs and taskforces set up by WHO and the United States Agency for International Development. Second, pharmacovigilance programmes are established to monitor the safety of marketed medicines by drug regulatory agencies such as in the United States and Europe or the WHO Prequalification of Medicines Programme, often used in less developed countries. Third, there has been investment in human resources to visually identify SF medicines, as well as in technical innovations such as simple and inexpensive tests (e.g. Minilab, which includes visual inspection, disintegration, colour reaction, and thin-layer chromatography) or mobile authentication services (e.g. Sproxil). Yet the problem remains quite relevant, and more needs to be done to combat the problem of SF medicines at a country level and internationally, including facilitating cooperation across agencies and harmonising policy efforts.

In Nigeria, our country of focus, the pharmaceutical market is valued at USD 4.5 billion and growing at over 9% annually [11]. Nigeria is highly import-dependent, importing more than 70% of finished products and producing the rest locally [12]. It is also almost 100% dependent on other countries for active pharmaceutical ingredients (API) needed for local manufacturing [13]. Starting in the 2000s, the National Agency for Food and Drug Administration and Control (NAFDAC) aggressively fought against SF medicines in the country, substantively reducing the prevalence of SF medicines, especially for antimalarials [14]. Some of NAFDAC’s actions included recommendations for changes in the law, dismissal of corrupt NAFDAC personnel, guidelines for NAFDAC staff behaviour and use of incentive schemes, destruction of large quantities of falsified and expired products, strict enforcement of registration guidelines, implementation of new guidelines to ensure that imported medicines are genuine, as well as raising public awareness [15,16]. In Nigeria, analgesics make up the largest share of the market (25%), followed by antibiotics (15%), multivitamins (15%), antimalarials (14%), and antihypertensives (8%) [17].

The consequences of SF medicines could be enormous in terms of health and socio-economic impact [1]. SF medicines could be harmful to patients – they could have adverse effects due to incorrect active ingredients (e.g. toxicity), they could fail to cure the disease, and they could cause prolonged illness and preventable deaths. Moreover, they could lead to the progression of antimicrobial resistance (AMR) [18–20]. From a health systems perspective, SF medicines could lead to additional care and thus increased out-of-pocket expenditures, burden on health care providers, and loss of confidence in the health system. Due to prolonged illness and a higher burden of disease, SF can also cause income loss for patients and productivity for businesses and the wider economy, contributing to increased poverty. Despite estimates on the health and economic impacts of SF medicines that would be useful for regulators to understand the gravity of the problem and make recommendations, there is still a gap in the knowledge, as the existing models mainly assess the impact of antimalarials and antibiotics [21].

The empirical evidence mainly focuses on SF medicines with inadequate API, such as lower than required or no API [4]. An exception is a recent literature review that displays the frequency of six reported issues concerning the quality of tested medicines, including an excessive amount of API (12% in 2019) [22]. However, we argue that too much API is as much of a concern as less or no API, and we should pay more attention to it. An API above the limit could be toxic, leading to the patient interrupting treatment prematurely. If the infection is not cleared, it could lead to an increased risk of transmission of a resistant strain [20]. AMR occurs when bacteria, viruses, fungi, and parasites change over time and no longer respond to medicines, making infections harder to treat and increasing the risk of disease spread, severe illness, and death [23]. In the case of antimicrobials, the success of newly emergent resistant strains depends on the level of API in the medicine. It is possible that an excess API could lead to a series of events that could contribute to AMR. For example, SF medicines with more than 100% API could still successfully kill all target pathogens but increase the selection among the bystander pathogens. Bystander selection is defined as the inadvertent pressure imposed by the treatment on microbes other than the targeted pathogen [24]. This would reduce resistance for the target pathogen but increase it for the bystander. Even though bystander selection is still understudied, and there is a need for more research to quantify its contribution to AMR, there is consensus that it is also a major factor contributing to AMR, for example, for antibiotics [24–26].

We present evidence from an ongoing research study in Nigeria, as part of which we tested 246 medicine samples, including analgesics (45%), antimalarials (27%), antibiotics (15%), and antihypertensives (13%). Among all medicines, 62 (25%) were found to have API outside the range (90–110%) as tested by high-performance liquid chromatography, suggesting that the prevalence of SF medicines is still quite high in the country. Among those who did not pass the laboratory testing, 35% (n = 22) were antihypertensives, 31% (n = 19) were analgesics, 19% were antibiotics (n = 12), and 15% (n = 9) were antimalarials. Most of these drugs (76%, n = 47) had two APIs. Further, 65% (n = 40) of drugs failed because at least one (or only one) ingredient had an API exceeding the limit. Among these, 50% (n = 31) of medicines with two ingredients failed because one was below and one was above the range, and 15% (n = 9) failed because one or two APIs in the medicines exceeded the 110% limit. The rest (35%, n = 22) failed because of their API being too low (**Table 1**).

**Table 1:** Prevalence of SF medicines by category and ingredients*

				Failed (one ingredient)		Failed (two ingredients)		
**Medicine category**	**Tested**	**Passed**	**Failed**	**Low API**	**High API**	**Low/high API**	**Low API**	**High API**
Analgesics	110 (45)	91 (83)	19 (17)	0 (0)	2 (11)	5 (26)	11 (58)	1 (5)
Antimalarials	67 (27)	58 (87)	9 (13)	0 (0)	0 (0)	5 (56)	1 (11)	3 (33)
Antibiotics	38 (15)	26 (68)	12 (32)	10 (83)	2 (17)	0 (0)	0 (0)	0 (0)
Antihypertensives	31 (13)	9 (29)	22 (71)	0 (0)	1 (5)	21 (95)	0 (0)	0 (0)
Total	246 (100)	184 (75)	62 (25)	10 (16)	5 (8)	31 (50)	12 (19)	4 (7)

API – active pharmaceutical ingredients

*Values are presented as n (%).

While any medication formulation may be considered substandard if it has too little or too much API compared to the formulation specification, more should be done to ensure the industry has and respects the standards. Excess API indicates some basic lack of expertise or wrong practice of overage in manufacturing. The local manufacturing of medicines across Africa has improved significantly, but efforts by the regulators are indispensable in ensuring continuous improvement and the protection of citizens’ public health.

These data present a limited picture of the current situation on a selected medicine sample in Nigeria. However, these findings suggest that governments should support local pharmaceutical companies in Africa investing in facilities and quality management systems to comply with good manufacturing practices [27]. Improving the quality of medicines and ensuring the correct amount of APIs – not too little, but not too much – would help limit further negative consequences of the emergence of AMR. In addition to better estimating the extent of this problem across different medicines and other countries, improving pharmaceutical quality assurance, and testing the impacts of health interventions to mitigate the risks associated with SF medicines, further research should investigate the impact of SF medicines with excessive APIs on AMR.
