# Erratum: Maffioli EM, Montás MC, Anyakora C. Excessive active pharmaceutical ingredients in substandard and falsified drugs should also raise concerns in low-income countries. J Glob Health. 2024 Jun 7;14:03029.

**DOI:** 10.7189/jogh.14.1403029err1

**Published:** 2024-12-20

**Authors:** Journal of Global Health Editorial Team



Following the publication of the manuscript ‘Excessive active pharmaceutical ingredients in substandard and falsified drugs should also raise concerns in low-income countries’ on 7 June 2024, the authors noted errors in the data presented in Table 1 and the preceding text regarding the numbers and percentages of analgesics with two ingredients that had failed lab testing. Specifically, the numbers and percentages for low/high API and low API were reversed for analgesics with two ingredients that failed lab testing, resulting in different totals. This error also caused changes in the key messages that are only present in the PDF version of the article. We detail these changes below.

Initial text: ‘76% (n = 47) of these medicines had two APIs. Further, 74% (n = 46) failed because at least one (or only one) ingredient had API exceeding the limit. Among these, 60% (n = 37) of medicines with two ingredients failed because one was below and one was above the range, and 14% (n = 9) failed because one or two APIs in the medicines exceeded the 110% limit. The rest (26%, n = 16) failed because the API was too low (**Table 1**).’

Corrected text: ‘Most of these drugs (76%, n = 47) had two APIs. Further, 65% (n = 40) of drugs failed because at least one (or only one) ingredient had an API exceeding the limit. Among these, 50% (n = 31) of medicines with two ingredients failed because one was below and one was above the range, and 15% (n = 9) failed because one or two APIs in the medicines exceeded the 110% limit. The rest (35%, n = 22) failed because of their API being too low (**Table 1**).’

Initial values in Table 1, row 3, label ‘Analgesics’: low/high API = 11 (58), low API = 5 (26).

Corrected in Table 1, row 3, label ‘Analgesics’: low/high API = 5 (26), low API = 11 (58).

Initial values in Table 1, row 7, label ‘Total’: low/high API = 37 (60), low API = 6 (10), high API = 4 (6).

Corrected values in Table 1, row 7, label ‘Total’: low/high API = 31 (50), low API = 12 (19), high API = 4 (7).

Initial key message in PDF: In Nigeria, 74% of the drugs that did not pass lab testing failed because of excessive active pharmaceutical ingredients.

Corrected key message in PDF: In Nigeria, 65% of the drugs that did not pass lab testing failed because of excessive active pharmaceutical ingredients.

The authors regret these errors, note that they do not affect the message of their viewpoint, and apologise for any inconvenience they may have caused.

These corrections have been made to the online and the PDF version of the manuscript on 20 December 2024, following the authors’ notice on 10 December 2024.

